# Functional Proteomics and Biological Screening of Protein Misfolding Under ER Stress Conditions in a Neuroblastoma Cell Model

**DOI:** 10.3390/cimb48080772

**Published:** 2026-07-29

**Authors:** Adele Serra, Elva Morretta, Michela Pecoraro, Maria Chiara Monti, Maria Pascale, Silvia Franceschelli

**Affiliations:** 1 Department of Pharmacy, University of Salerno, Via Giovanni Paolo II, 84084 Fisciano, SA, Italy; adserra@unisa.it (A.S.); elva.morretta@unina.it (E.M.); mariachiara.monti@unina.it (M.C.M.); pascale@unisa.it (M.P.); 2 Ph.D. Program in Drug Discovery and Development, University of Salerno, 84084 Fisciano, SA, Italy; 3 Department of Pharmacy, University of Naples, Federico II, Pharmacy, 80131 Naples, Italy

**Keywords:** ER stress, proteomics, neurodegeneration, DARTs, protein misfolding

## Abstract

Endoplasmic reticulum (ER) stress represents a critical pathophysiological condition that plays a central role in the development of various human diseases, including protein misfolding diseases. While the small molecule Vx-445 (Elexacaftor) exhibits robust cellular bioactivity, its cryptic intracellular targets and off-label mechanisms of action remain poorly defined. This study investigates the cytoprotective efficacy and molecular targets of Vx-445 in Thapsigargin-induced ER stress in a neuronal cell model. Integrating biochemical assays, gene expression proteomics, and label-free functional proteomics, we demonstrate that Vx-445 significantly mitigates oxidative stress by reducing intracellular levels of reactive oxygen species, restores calcium homeostasis to baseline levels, and prevents apoptosis by inhibiting cytochrome c release. These phenotypic modifications correlated with changes in proteomic expression and were validated by Drug Affinity Responsive Target Stability (DARTS) analysis to map restored cellular pathways and identify potential protein interaction partners. Together, these findings uncover alternative molecular targets for Vx-445, providing a mechanistic basis for drug repurposing strategies in endoplasmic reticulum stress-related diseases.

## 1. Introduction

Proteins are essential biological macromolecules for all living organisms. A critical requirement for their proper function is correct folding, which results in a specific and thermodynamically stable conformation [1]. These macromolecules are designed to perform a wide range of functions within the complex cellular environment, making the structure–function relationship fundamental. In eukaryotic organisms, the primary site for protein synthesis and folding is the endoplasmic reticulum (ER), which also plays a critical role in lipid and carbohydrate production and serves as a primary reservoir for Ca^2+^ ions [2].

Cellular homeostasis depends strictly on an optimal ER environment for proper protein folding and quality control, particularly in secretory cells with high protein demand, such as plasma cells, pancreatic β-cells, or hyperproliferative malignant cells. Protein synthesis, folding, function, and degradation are governed by the “proteostasis network” (PN), an intricate system that maintains proteome equilibrium under physiological conditions [3] continuously modulated by mutations, epigenetic modifications, environmental stress, and ageing, which constantly challenges the cell’s protein processing capacity [4].

Protein folding within the ER is a complex, multistep process that requires a tightly regulated oxidative environment to facilitate disulfide bond formation. Since oxidative stress is closely related to ER stress, increased demand for protein folding leads to increased reactive oxygen species (ROS) in the ER. Under pathological conditions, excess ROS production disrupts the balance between ROS levels and antioxidant mechanisms, compromising antioxidant defenses and triggering oxidative stress [5]. Intracellular Ca^2+^ signaling networks dictate critical cellular decisions, directly influencing survival and apoptosis. Because the ER serves as the primary intracellular Ca^2+^ reservoir, disruption of homeostasis directly provokes ER stress. The lumen of the ER houses essential Ca^2+^-dependent chaperones—including glucose-regulated protein 94 (GRP94), BiP (GRP78), and calreticulin—whose folding fidelity depends on high luminal Ca^2+^ concentrations [6]. Moreover, the depletion of ER Ca^2+^, paired with cytosolic Ca^2+^ overload, triggers apoptotic pathways, leading to cell death [7]. This homeostatic collapse extends beyond the ER to affect downstream organelles, particularly the mitochondria. Recent studies have revealed not only functional interactions but also direct physical connections between the ER and mitochondria at specialized contact sites known as mitochondria-associated membranes (MAMs). These connections coordinate mitochondrial homeostasis, Ca^2+^ signaling, lipid metabolism, autophagy, and apoptosis [8]. Consequently, the accumulation of misfolded proteins in the ER can lead to mitochondrial dysfunction [9].

Under ER stress conditions, the cell rapidly activates the unfolded protein response (UPR) to restore homeostasis. In mammalian cells, each distinct branch of the UPR produces a specific basic leucine zipper transcription factor (bZIP) which, through homodimerization or heterodimerization, dictates cell fate [10]. The three main UPR sensors are inositol-requiring enzyme 1 (with isoforms IRE1α/β), double-stranded RNA-activated protein kinase (PKR)-like ER kinase (PERK), and activated transcription factor 6 (with isoforms ATF6α/β in mammals). Under physiological conditions, these proteins are maintained in an inactive state through their stable association with BiP, a key ER chaperone [11]. However, the accumulation of unfolded or misfolded polypeptides creates a competitive binding environment. As BiP preferentially binds to these aberrant proteins, this structural dissociation relieves sensor inhibition, initiating downstream UPR signaling cascades. A variety of cell-intrinsic and environmental factors can perturb protein processing, culminating in protein misfolding diseases (PMDs) that encompass genetic, cardiovascular, neurodegenerative, metabolic, autoimmune, and fibrotic disorders, as well as viral infections and cancer [12]. Notably, in numerous genetic conditions, point mutations do not result in the complete loss of protein expression. Instead, they lead to the synthesis of structurally aberrant variants that fail to pass quality control, resulting in premature degradation or mistargeting away from their intended cellular destination. Cystic fibrosis (CF) is an example of this phenomenon. It is an autosomal recessive disorder caused by loss-of-function mutations in the gene encoding the cystic fibrosis transmembrane conductance regulator (CFTR) protein. The most common mutation, occurring in 85% of patients, is a 3 bp in-frame deletion that eliminates phenylalanine at position 508, Phe508del. In CF, this mutation disrupts post-translational processing and trafficking, drastically reducing its half-life and preventing functional ion channel expression at the cell surface [13].

Historically, therapeutic strategies for CF focused on managing damage to individual organs affected by CFTR mutations. However, the advent of CFTR modulators, a novel class of drugs, fundamentally shifted the treatment paradigm from symptom management to targeting the underlying molecular defect, drastically improving patient outcomes. In vitro studies demonstrate that these small-molecule drugs directly engage specific structural domains of CFTR during or after its biosynthetic processing. This targeted interaction facilitates correct polypeptide folding, effectively overriding the structural defects imposed by the mutation. These therapeutic small molecules are classified into distinct functional categories based on their mechanism of action, including potentiators, correctors, read-through agents, amplifiers, and stabilizers [14]. Such highly effective therapies have transformed the lives of patients and their long-term prognosis. Currently, one potentiator (Ivacaftor) and three correctors (Lumacaftor, Tezacaftor, and Elexacaftor) are in clinical use [15].

This work focuses on correctors, also referred to as pharmacochaperones, which enhance the amount of CFTR protein in the plasma membrane. Lumacaftor (Vx-809) and Tezacaftor (Vx-661) are structural analogs that belong to the same distinct cluster classified as type I correctors, and both share a 1,3-Benzodioxol-5-yl-Cyclopropane Carboxamide (BCC) head group. By contrast, Elexacaftor (Vx-445) has a different chemical structure and is classified as a type III corrector [16,17]. Despite significant progress, the precise mechanism of action of these drugs remains to be fully elucidated.

Over time, small-molecule CF correctors have demonstrated therapeutic potential, extending far beyond their originally intended application. Pecoraro and co-workers [18] were the first to demonstrate that Vx-809 not only corrects the misfolding responsible for CF but also operates via a broader pharmacological mechanism, mitigating the protein misfolding responsible for ER stress. Specifically, their study revealed that Vx-809 restores homeostasis in cell models unrelated to CF, including lung adenocarcinoma (A549) and melanoma (A375) cells, following induction of ER stress with Thapsigargin (TG), a selective inhibitor of sarcoplasmic and ER Ca^2+^ ATPase (SERCA). SERCA inhibition depletes ER Ca^2+^ stores, elevates cytosolic Ca^2+^ levels, and activates ER stress-related signaling. VX-809 was found to exert a pleiotropic effect, not only on the main UPR sensors but also on pathways such as oxidative stress, inflammation, apoptosis, and Ca^2+^homeostasis. As further support for the potential of Vx-809 in the treatment of PMDs, Pecoraro and colleagues further reported its neuroprotective effects in SH-SY5Y neuronal cells, a well-established in vitro model for neurodegenerative disorders, under ER stress conditions. In this model, Vx-809 similarly reduced ER stress and its associated pathological process, validating its cytoprotective capacity in a neuronal lineage [19].

Despite the clinical efficacy of pharmacological correctors, the ambiguity surrounding their molecular interactome under physiological conditions remains a major obstacle [16,20,21]. While Vx-809 has been reported to modulate ER stress through broad interactions with protein partners [18,19], the cellular-independent activities of the type 3 corrector Vx-445 [22] remain largely unexplored.

In this study, we diverge from canonical applications to investigate the CFTR-independent cytoprotective efficacy of Vx-445. Employing a multidisciplinary framework in SH-SY5Y neuronal cells, we integrated biochemical assays for ROS and Ca^2+^homeostasis with advanced expression and functional proteomics (DARTS).

Through this comprehensive approach, we sought to systematically characterize the off-target mechanisms of VX-445. Our findings not only identify novel intracellular protein interaction partners but also establish a clear mechanistic rationale for the pharmacological repositioning of this compound to treat ER stress-driven pathologies.

## 2. Materials and Methods

### 2.1. Reagents

Vx-445 (S8851) was purchased from Selleckchem (Houston, TX, USA). The monoclonal antibodies used included anti-Cytochrome c (66264-1-Ig, Proteintech, San Diego, CA, USA) and anti-GAPDH (sc-32233, Santa Cruz Biotechnology, Heidelberg, Germany). Secondary antibodies (anti-mouse, A90-137P) were acquired from Bethyl Laboratories (Montgomery, TX, USA). Thapsigargin, H_2_DCF-DA, Ionomycin, and FURA 2AM were all purchased from Sigma-Aldrich (St. Louis, MO, USA).

### 2.2. Cell Culture

In 75 cm^2^ sterile culture flasks, a human neuroblastoma cell line (SH-SY5Y, CL0208, Elabscience, Houston, TX, USA) was grown in culture once a week using Dulbecco’s modified Eagle’s Medium/F12 (DMEM/F12; Euroclone, Milan, Italy) containing 10% Fetal Bovine Serum (FBS; Euroclone, Milan, Italy), 100 U/mL of penicillin, and 100 μg/mL of streptomycin in a humidified atmosphere with 5% CO_2_ at 37 °C. Less than 80% of the cells were always confluent during the experiments.

### 2.3. Experimental Protocol

To create an ER stress situation, the cells were seeded and then pretreated with 300 nM Thapsigargin (TG) for 15 and 30 min, 1 h, and 2 h after 24 h of adhesion. Following the removal of TG, cells were then provided with new media, both with and without Vx. After a 24 h treatment with corrector Vx-445 (3 µM), cell lysates were obtained from both the Vx-treated and TG-treated cells.

### 2.4. Intracellular ROS Detection

Neuroblastoma cells (8 × 10^4^ cells/well), seeded into 24-well plates and treated as shown above, were used to assess intracellular ROS production. After treatment, cells were collected, given two PBS washes, and then incubated for 30 min at 37 °C in the dark in PBS containing a 2′,7′-dichlorofluorescin diacetate (H_2_DCF-DA; 10 μM) probe. Fluorescence-activated cell sorting (FACScan; Becton-Dickinson, San Jose, CA, USA) was subsequently employed to measure the fluorescence of the cells, and Cell Quest software version 4.1 as used to analyse the results.

### 2.5. Intracellular Calcium Content Analysis

A membrane-permeant acetoxymethyl ester version of Fura 2, Fura 2-AM, a fluorescent indicator dye, was used to assess intracellular Ca^2+^concentrations. In 24-well tissue culture plates, SH-SY5Y cells (3 × 10^4^ cells/plate) were seeded and subjected to the previously mentioned treatment.

Cells were rinsed with phosphate-buffered saline (PBS) at the end of the treatment, reconstituted, and incubated for 45 min in 1 mL of Hank’s balanced salt solution (HBSS), which contained 5 μM Fura 2-AM.

Afterward, cells were moved to a cuvette for spectrofluorometer measurement (Perkin-Elmer LS-55, Milano (MI), Italy), and excess Fura 2-AM was removed.

Subsequently, the cuvette was filled with Ionomycin (1 μM), a Ca^2+^ ionophore commonly used to investigate the role of Ca^2+^ in specific cellular processes, in Ca^2+^-free HBSS/0.5 mM EGTA solution. When the excitation wavelength was changed from 340 to 380 nm, emission fluorescence was observed at 515 nm. According to Pecoraro and co-workers [19], there was a considerable correlation between intracellular free Ca^2+^ and the fluorescence intensity ratio of 340/380 nm (F340/F380). Measured and reported as a delta (Δ) rise in the fluorescence ratio (F340/F380 nm), Ionomycin-induced increases in this ratio were seen.

### 2.6. Extraction of Proteins and Western Blot

As described in the experimental protocol, neuroblastoma cells (1 × 10^6^ cells/plate) were plated and processed. Lysis buffer (150 mM NaCl; 20 mM Tris-HCl, pH 7.5; 1 mM EGTA; 1 mM Na_2_EDTA; 1% sodium deoxycholate; 2% IGEPAL; 1X protease and phosphatase inhibitor cocktail) was added to the cells for 30 min to extract the proteins. The lysates were then centrifuged for 15 min at 4 °C and 14,000 rpm. A total of 60 µg of protein was loaded into 12% acrylamide gels, separated by SDS-PAGE under denaturing conditions, and then transferred to nitrocellulose membranes using a minigel instrument (Bio-Rad Laboratories, Richmond, BC, Canada) after the Bradford methodology was used to determine the protein content.

After blocking for two hours at room temperature in 5% Tris-buffered saline in non-fat dry milk, blots were incubated overnight at 4 °C with primary antibody against Cytochrome C. The loading control was GAPDH. The relevant secondary antibodies were similarly added for one hour at room temperature following different PBS/0.1% Tween washes. Enhanced chemiluminescence (ECL) reagents and Western blot imaging (LAS 4000; GE Healthcare, Milan, Italy) were used to visualize the immunoreactive protein bands. ImageJ software Version 1.54j was used to quantify the results of Western blot imaging.

### 2.7. Expression Proteomics

SH-SY5Y cells were treated to simulate ER stress conditions. The cells were seeded and then pretreated with 300 nM TG for 2 h after 24 h of adhesion. After washing off the TG, the cells were provided with fresh culture medium, either with or without Vx. After a 24 h treatment period with corrector Vx-445 (3 µM), the cells were detached with trypsin and, after two washes with PBS, the pellet was subjected to functional proteomic analysis.

The obtained cell pellets were lysed in an ammonium bicarbonate (AmBic, 50 mM, pH 8.5) buffer comprising 8 M Urea, 0.5% w/vol sodium deoxycholate, and a protease inhibitor cocktail (1× final concentration). Lysis was achieved through sonication (Branson Digital Sonicator, 30% amplitude, on/off pulses of 9.9 s, total lysis time of 1 min), followed by sample centrifugation at 21,000× *g* and 18 °C for 20 min. The protein concentration of the supernatants was evaluated using the Bradford assay. Equal protein amounts (500 µg in 90 µL) were then submitted to an in-solution digestion protocol with a LysC/trypsin mixture, as reported elsewhere [23]. The obtained peptide mixtures were then dried under vacuum, dissolved in 5% FA, and desalted through Sep-Pak C18 1 cc (50 mg) cartridges (Waters, Milford, MA, USA). Briefly, the cartridges were activated by flushing 3 mL of 100% CH_3_CN and then conditioned with 3 mL of 0.1% FA. Samples were then loaded, desalted by flushing the cartridges with 3 mL of 0.1% FA, and finally eluted by flushing with 500 μL of 80% CH_3_CN, 20% H_2_O, and 0.1% FA two times. For the subsequent MS analysis, the peptide mixtures were dried under vacuum and re-dissolved in 10% FA. Subsequently, 1 μg of each digest was analyzed through nano-UHPLC-MS/MS on a Tribrid Orbitrap Mass Spectrometer coupled to a *Vanquish Neo* UHPLC system (ThermoFisher Scientific, Bremen, Germany) equipped with an EASY-Spray PepMAP^TM^ Neo C18 column (1500 bar, 2 μm, 100 Å, 75 μm × 750 mm, ThermoFisher Scientific, Bremen, Germany). Peptide elution was achieved at a flow rate of 300 nL/min with the following gradients: 0.1 min at 1% B, 0.1 min to 180.1 min to 45% B, 180.2 min to 99% B, then held at 99% B for 16 min and brought back to 1% B (A: H_2_O, 0.1% FA; B: 80% CH_3_CN, 20% H_2_O, 0.1% FA). The mass spectrometer was operated in data-dependent acquisition mode, with a cycle time of 1.5 s. Full-scan MS spectra were acquired in the Orbitrap with the following settings: a scan range of 375–1500 *m*/*z*, normalized full-scan automatic gain control (nAGC) target of 100% at 240,000 resolution, and a maximum injection time of 50 ms. Precursors were isolated in the Quadrupole, and MS2 spectra were generated with HCD (normalized collision energy of 30%) and acquired in the Ion Trap (rapid scan mode), with an nAGC target of 300% and a maximum injection time of 20 ms. The obtained raw files were then analyzed through *Proteome Discoverer* (version 3.1.0.638). Spectra were searched by Sequest against a reviewed Homo sapiens database (UP000005640, March 2024, gene count 20,656) using the following parameters: trypsin digestion; a maximum of two missed cleavages; cysteine carboxyamidomethylation as fixed modification, and methionine oxidation as variable modification. Precursor mass and fragment mass tolerance were, respectively, set at 10 ppm and 0.6 Da. Label-free quantification was performed using both unique and razor peptides, and protein abundance ratios were calculated through a pairwise ratio-based approach, corroborated by a background-based t-test.

### 2.8. Drug Affinity Responsive Target Stability (DARTS)

SH-SY5Y cell pellets were lysed in M-PER (Mammalian Protein Extraction Reagent, Thermo Scientific, Waltham, MA, USA) supplied with a protease inhibitor cocktail (Gene-Spin, Milan, Italy), as reported by the manufacturer. The lysate was centrifuged at 10,000× *g* for 10 min at 4 °C, and the Bradford assay (Bio-Rad, Hercules, CA, USA) was used to determine protein concentrations of the obtained supernatant. Equal protein aliquots (80 μL at 3 μg/μL) were then incubated with DMSO or with increasing amounts of Vx-445 (final concentrations of 1 M, 10 M, and 100 M) for 1 h at room temperature and under continuous shaking. The final DMSO amount was 1.7% *vol*/*vol* in each sample. Samples were then submitted to limited proteolysis (1:1000 *w*/*w* subtilisin to proteins ratio) for 30 min (25 °C, 450 rpm), except for an aliquot of the DMSO-treated lysate, which was submitted to mock proteolysis. Subtilisin was then quenched with 1 mM phenylmethane sulfonyl fluoride (PMSF, Sigma Aldrich-Merck, Milan, Italy). This experiment was performed in triplicate.

Then, 20 μg of each sample were heated at 95 °C in Laemmli buffer, and proteins were separated through 1D-SDS-PAGE on 4–12% polyacrylamide gradient gels (Criterion^TM^ XT Precast Gel, 4–12% Bis-Tris, Bio-Rad, Hercules, CA, USA). The gels were Coomassie stained (Coomassie Brilliant Blue R-250, Bio-Rad, Hercules, CA, USA), and gel bands were excised and submitted to an in situ tryptic digestion protocol. Peptide mixtures were then dried under vacuum and dissolved in 10% formic acid (FA) for the subsequent nano-UHPLC-MS/MS analysis. A total of 1 μL of each digest was analyzed on an Orbitrap Q-Exactive Classic Mass Spectrometer coupled to an UltiMate 3000 Ultra-High Pressure Liquid Chromatography (UHPLC) system (ThermoFisher Scientific, Bremen, Germany) equipped with an EASY-Spray PepMAP^TM^ RSLC C18 column (3 μm, 100 Å, 75 μm × 50 cm, ThermoFisher Scientific, Bremen, Germany). Peptide elution was achieved at a flow rate of 300 nL/min with the following gradients: 1 min at 3% B, 1 min to 40 min to 28% B, 40 min to 41 min to 70% B, 41 min to 49 min at 70% B and 50 min back to 3% B until 60 min (A: 95% H_2_O, 5% CH_3_CN, 0.1% AcOH; B: 95% CH_3_CN, 5% H_2_O, 0.1% AcOH). The mass spectrometer was set in the data-dependent acquisition mode. Full scan MS spectra were acquired as follows: scan range 375–1500 *m*/*z*, full-scan automatic gain control (AGC) target 3e6 at 70,000 resolution, maximum injection time 50 ms. MS2 spectra were generated for up to 8 precursors (normalized collision energy of 28%), and the fragment ions were acquired at a resolution of 17,500, with an AGC target of 1 × 10^5^ and a maximum injection time of 80 ms. The obtained raw files were analyzed through *Proteome Discoverer* (version 2.4.1.15). *MSPepSearch* was used to perform a spectral library search (NIST Human Orbitrap HCD Library) with a mass tolerance of 10 ppm for MS1 and 0.02 Da for MS2. The target False Discovery Rate (FDR) was set to 1% (strict) or 5% (relaxed). Subsequently, MS/MS spectra were also searched by Sequest against the SwissProt Homo sapiens database as follows: trypsin digestion; maximum of two missed cleavages; cysteine carbamidomethylation as fixed modification; methionine oxidation, protein N-terminal acetylation and/or demethylation as variable modifications; precursor mass tolerance of 10 ppm and fragment mass tolerance of 0.02 Da. Label-free quantification was performed on both unique and razor peptides, and a pairwise ratio-based approach was used to evaluate Vx-445 vs. CTRL protein abundances. For each calculated ratio, a background-based *t*-test was performed.

### 2.9. Statistics for Analysis

Data evaluations and statistical analysis were conducted using the commercially available program GraphPad Prism8 (GraphPad Software Inc., San Diego, CA, USA). The information is displayed as the mean ± standard error of a minimum of three separate technically triplicate studies. The non-parametric Mann–Whitney U method was used to collect statistical data between the experimental points. The differences were considered significant if the *p*-values were within the range of less than 0.01 to 0.05.

## 3. Results

### 3.1. Vx-445 Fights Oxidative Stress Triggered by ER Stress Condition

Oxidative and ER stress are inextricably linked. Although both processes occur under physiological conditions, alterations in the cellular environment can exacerbate them, driving the pathogenesis of various human disorders. Oxidative stress can disrupt the delicate redox balance required within the ER lumen for the synthesis and folding of secretory proteins, leading to protein misfolding and subsequent ER stress. Importantly, factors that induce protein misfolding within the ER also increase ROS production. This reciprocal loop creates a self-amplifying cycle where each stress state continuously reinforces and exacerbates the other [24].

To assess the effect of the corrector on oxidative stress, we evaluated the total ROS production induced by TG, with or without Vx-445 administration, using cytofluorimetric analysis and employing the fluorescent probe H_2_DCF-DA.

**Figure 1:**
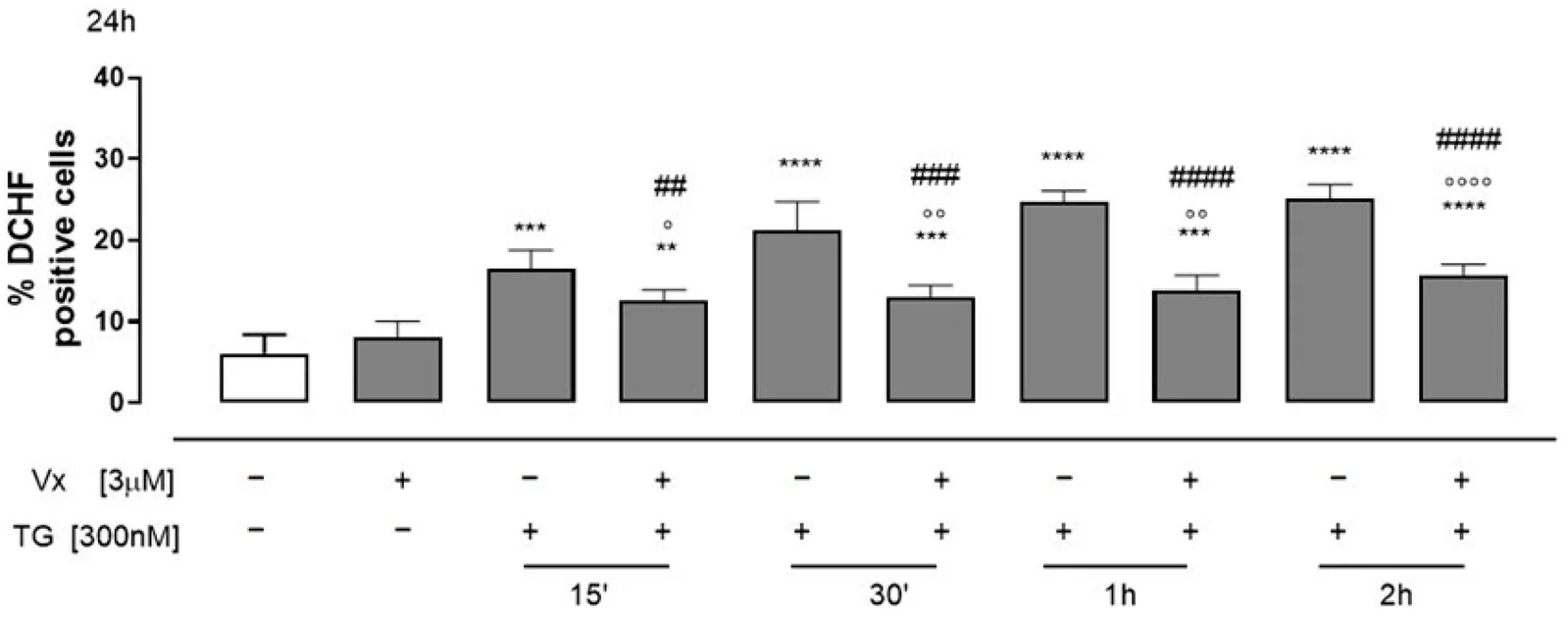
Vx-445 attenuates TG-induced oxidative stress. To induce ER stress conditions, cells were pretreated with 300 nM TG for different experimental times (15 min, 30 min, 1 h, and 2 h). The corrector Vx-445 (3 µM) was then administered for 24 h. The 2′,7′-dichlorofluorescein diacetate (H_2_DCF-DA) probe was used to measure the amount of ROS produced in SH-SY5Y cells. The percentage of DCHF-positive cells in at least three independent experiments, each conducted in triplicate, was used to express the mean ± SEM of ROS generation. Statistical significance was evaluated using the non-parametric Mann–Whitney U test. ** *p* < 0.005, *** *p* < 0.001, and **** *p* < 0.0001 vs. untreated cells; ° *p* < 0.05, °° *p* < 0.005, and °°°° *p* < 0.0001 vs. Vx-445 -treated cells; ## *p* < 0.005, ### *p* < 0.001, and #### *p* < 0.0001 vs. TG-treated cells.

This attenuation of oxidative stress was consistently observed across all experimental time points (Figure 1). The data demonstrate the robust antioxidant capacity of the corrector under reticular stress.

### 3.2. VX-445 Restores Intracellular Calcium Homeostasis and Replaces Luminal ER Stores

The ER serves as the primary intracellular Ca^2+^ reservoir, a sequestering capacity actively maintained by the Sarco/ER Ca^2+^-ATPase. Ca^2+^ plays a crucial role, not only in protein folding within the ER, supported by various Ca^2+^-dependent chaperones, but also as a key mediator of intracellular signaling in response to stimuli such as second messengers, kinases, and other modulators [12]. Given the critical importance of Ca^2+^ homeostasis and its wide-ranging effects on physiology, we investigated whether the Vx-445 corrector could modulate this pathway.

**Figure 2:**
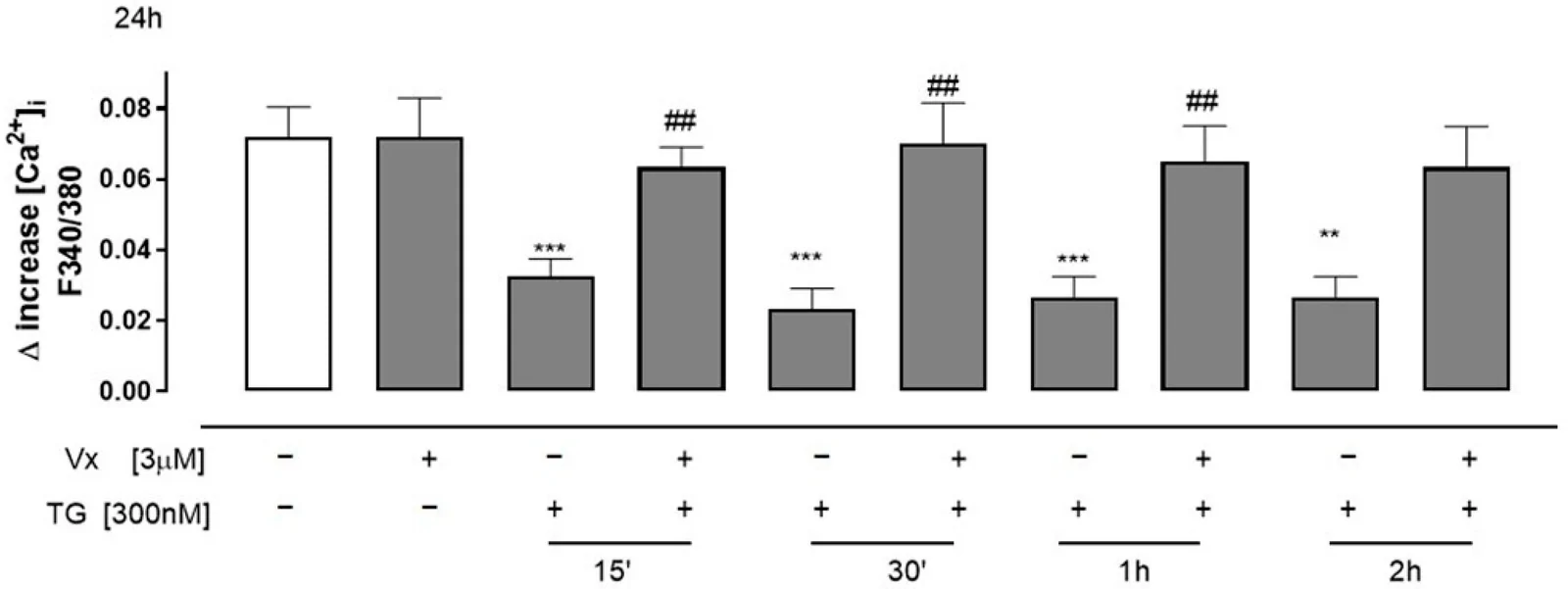
Vx-445 restores calcium homeostasis. To induce ER stress, SH-SY5Y cells were pretreated with 300 nM TG at different experimental times: 15 min, 30 min, 1 h, and 2 h. After that, Vx-445 (3 µM) was added for 24 h. The impact of VX-445 on total mobilizable intracellular Ca^+2^ stores was quantified under extracellular Ca^+2^-free conditions upon stimulation with the selective Ca^+2^ ionophore Ionomycin (1 μM). The results show the mean ± S.E.M. of the delta (Δ) increase in the fluorescence of the FURA 2 ratio (340/380 nm) from a minimum of three independent experiments, each carried out in triplicate. The findings are presented as the average ± standard error of triplicate data from a minimum of three separate and identical tests. Statistical variations were evaluated using the non-parametric Mann–Whitney U test. ** *p* < 0.005 and *** *p* < 0.001 vs. untreated cells; ## *p* < 0.005 vs. TG-treated cells.

The impact of Vx-445 on intracellular Ca^2+^ concentrations was assessed using FURA-2 AM in a Ca^2+^-free incubation medium. To evaluate total cytosolic Ca^2+^levels, Ionomycin (1 μM), a selective Ca^2+^ ionophore, was employed. The results (Figure 2) show that cells pretreated with TG and subsequently exposed to Vx-445 exhibited a significant increase (*p* < 0.001) in intracellular Ca^2+^levels compared with cells treated with TG alone. These findings suggest that a 24 h treatment with Vx-445 successfully restored intracellular Ca^2+^pools, effectively returning them to their initial physiological levels.

### 3.3. Vx-445 Prevents Apoptotic Signaling by Inhibiting Mitochondrial Cytochrome C Release

The ER maintains extensive interactions with nearby organelles, including mitochondria, phagosomes, endosomes, lysosomes, and the plasma membrane [25]. Its structural association with mitochondria is specifically mediated by MAM [26]. Notably, the accumulation of misfolded proteins during chronic ER stress has been shown to impair mitochondrial activity [27]. To investigate whether Vx-445 influences this process, cytochrome c (CytC) release was evaluated by Western blot analysis.

**Figure 3:**
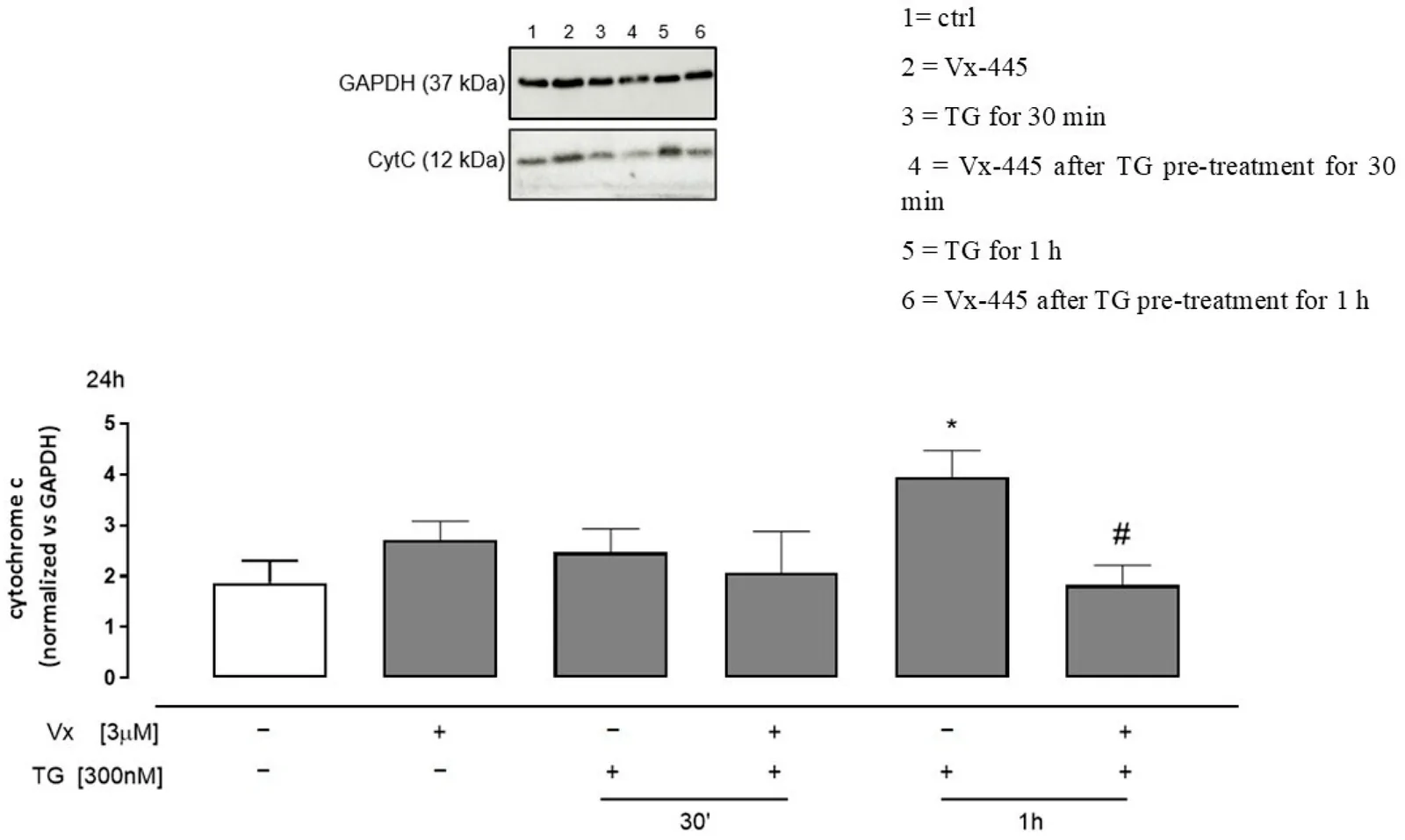
Vx-445 inhibits the mitochondrial release of CytC into the cytoplasm. Following the experimental plan, SH-SY5Y cells were pretreated with 300 nM TG at different experimental times: 30 min and 1 h to induce ER stress conditions. After that, Vx-445 (3 µM) was administered for 24 h. The expression of cytochrome C in the neuroblastoma cell line was evaluated by Western Blot assay. Lane assignments: 1 = ctrl; 2 = Vx-445; 3 = TG for 30 min; 4 = Vx-445 after TG pre-treatment for 30 min; 5 = TG for 1 h; 6 = Vx-445 after TG pre-treatment for 1 h. GAPDH protein expression was used as a loading control. Densitometric data, expressed as mean ± S.E.M, were derived from at least three independent biological replicates performed in triplicate. The Mann–Whitney U test analysis was performed on the data. * *p* < 0.05 vs. non-treated cells; # *p* < 0.05 vs. TG-treated cell.

According to the experimental timeline, TG was administered to induce ER stress in the SH-SY5Y cell line. Quantitative Western blot analysis revealed a robust increase in CytC release after just 1 h of TG pre-treatment, confirming the onset of the stress condition. Conversely, exposure to Vx-445 for 24 h reduced CytC levels, suggesting that Vx-445 may alleviate ER stress and indirectly contribute to the recovery of normal mitochondrial function, as depicted in Figure 3.

### 3.4. Vx-445 Attenuates the Expression of Proteins Exhibiting Oxidoreductase Activity Induced Under Cellular Stress Conditions

**Figure 4:**
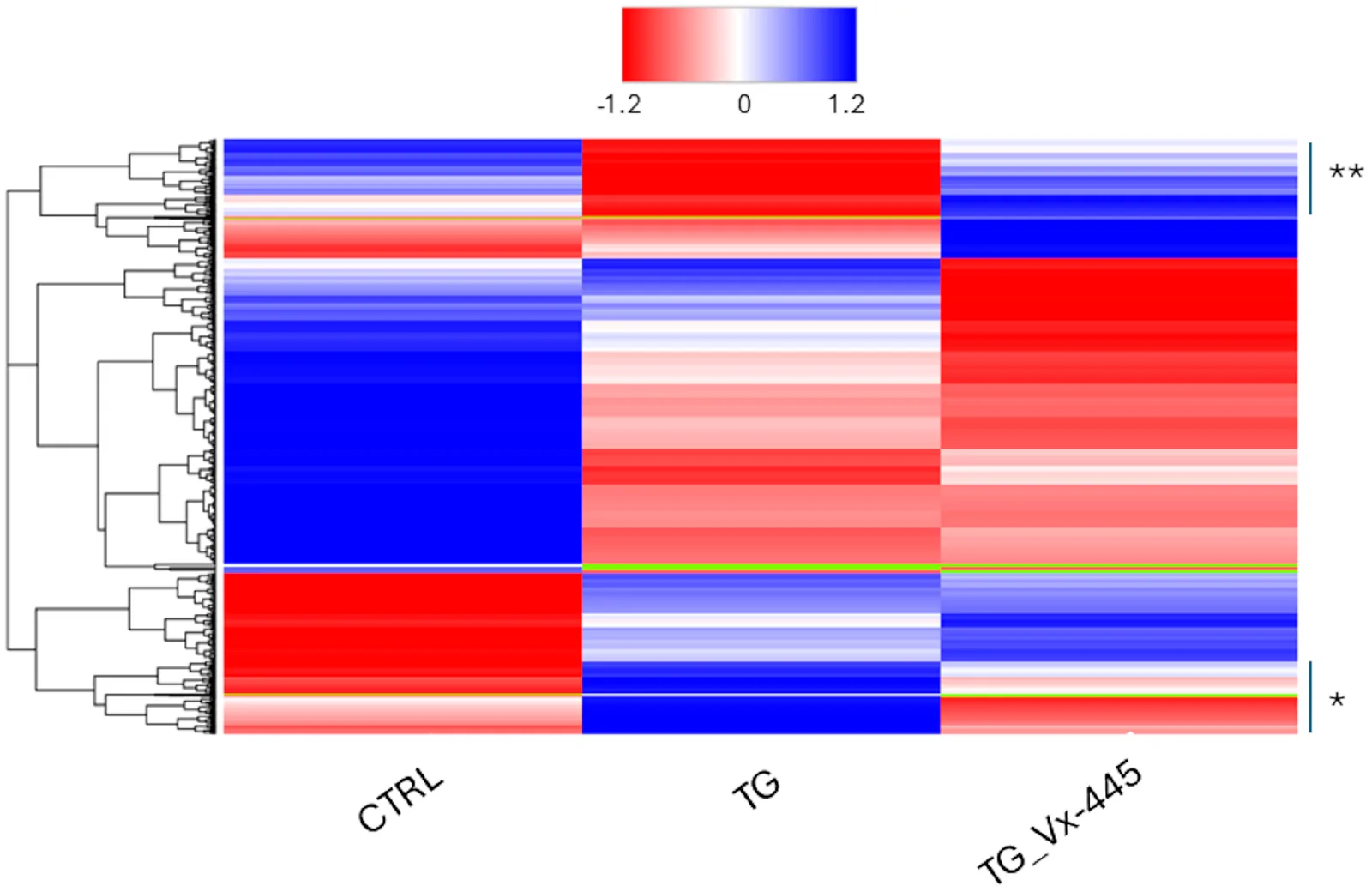
Hierarchical clustering of SH-SY5Y proteome shifts upon TG and TG-Vx-445 treatment compared to CTRL. The red and the blue colors in the heat map refer to the low- and high-abundance proteins, respectively. Asterisks highlight two highly responsive target segments displaying complete pharmacological reversal: the single-asterisk signature * contains proteins upregulated by TG and normalized (downregulated) by VX-445, while the double-asterisk signature ** encompasses proteins repressed by TG and subsequently restored (upregulated) by the corrector.

To comprehensively elucidate the molecular mechanisms underlying the effects of Vx-445 action on SH-SY5Y cells, we conducted a label-free quantification (LFQ) mass spectrometry-based expression proteomic analysis. The profiling focused on three distinct experimental groups derived from cell lysates of SH-SY5Y: control sample (CTRL), cells exposed to TG for 2 h (stress sample, TG), and cells sequentially treated with TG and Vx-445 for 24 h (corrector sample, TG_Vx-445). These samples were digested by trypsin and analyzed in parallel via nano UHPLC-MS/MS. Subsequently, a comprehensive bioinformatic analysis was deployed to compare abundance changes of the identified proteins across the experimental conditions. From a deep proteomic coverage comprising approximately 7500 identified and quantified protein groups, the resulting differentially expressed proteins (DEPs) were extracted and are visually resolved via the hierarchical clustering heat map displayed in Figure 4.

Proteomic analysis revealed substantial variations in protein expression profiles among the different populations. The box plots of ungrouped and grouped protein abundance distributions (Figure S1) demonstrated consistent medians and similar interquartile ranges across various experimental conditions. Furthermore, principal component analysis (PCA; Figure S2) reveals a clear distinction between the control, TG-treated, and TG-Vx-445-treated samples, with a good separation between conditions.

**Figure 5:**
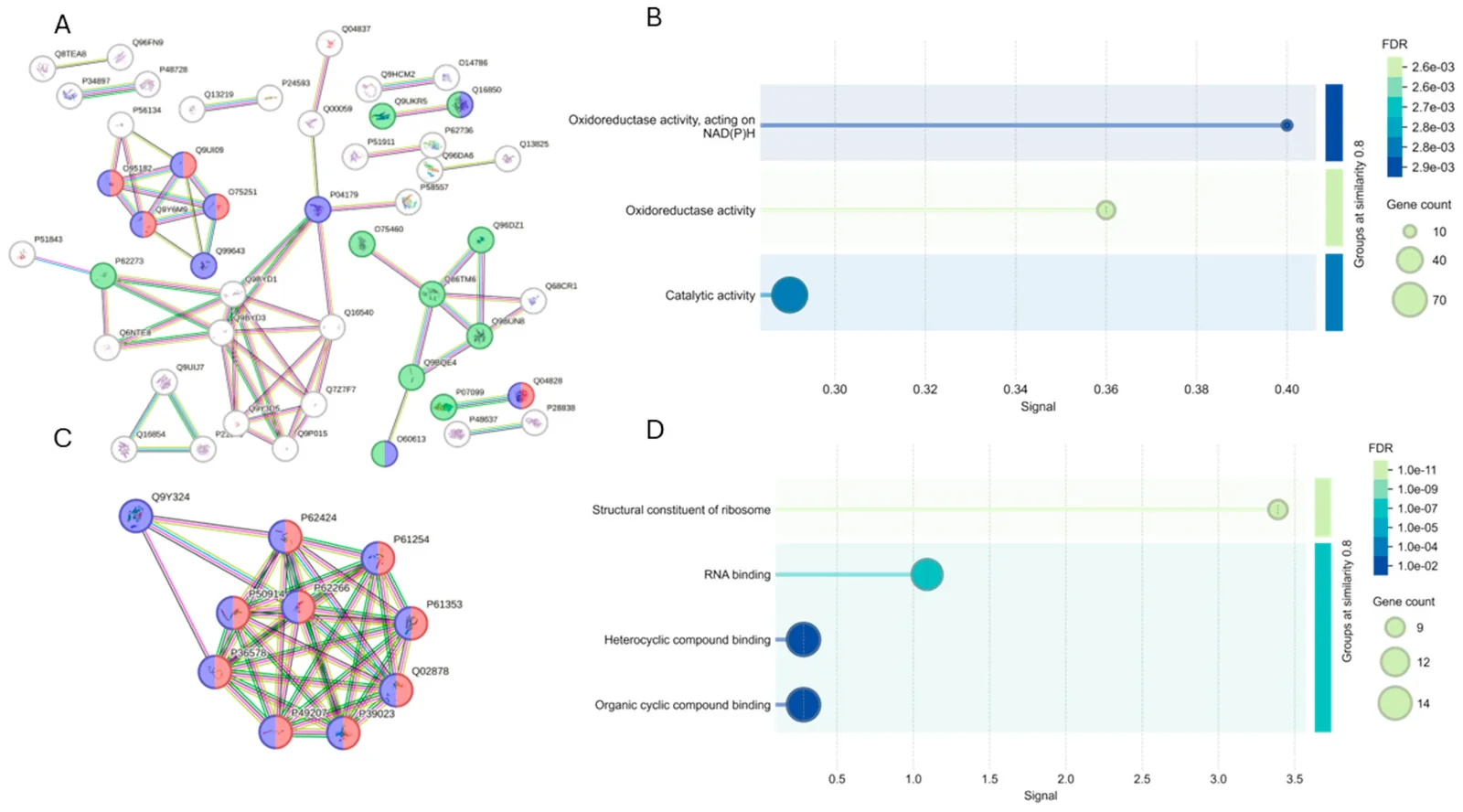
Cluster analysis of the proteins belonging to the (**A**,**C**) segments, constructed using the STRING software with a 0.7 confidence level. The disconnected nodes are hidden. Each protein is reported using its Uniprot code. Gene Ontology (GO) enrichment analysis by molecular function of the proteins belonging to the (**B**,**D**) segments, obtained using the STRING software. The order of appearance in the bar graph is related to enrichment of proteins in each biological pathway. The FDR value is also reported.

Hierarchical clustering of the expression profile (Figure 4) highlighted two distinct proteomic segments of therapeutic interest, denoted by stars: the one-star segment (*) comprises 175 proteins that were upregulated by TG, with respect to the control, and subsequently downregulated by the corrector Vx-445; conversely, the two-star segment (**) contains 20 proteins that are downregulated by TG in respect to the control and are upregulated by the corrector Vx-445. The proteins belonging to the * (175 hits) and ** (20 hits) segments were independently clustered using the software STRING, as shown in Figure 5.

The cluster and Gene Ontology analyses of the proteins belonging to the * segment (Figure 5A,B) revealed the presence of many hits belonging to the ER compartment (Figure 5A in green), as well as proteins endowed with oxidoreductase activity that were either NAD[P]H-dependent or independent (Figure 5A in blue and red, respectively, and Figure 5B). These proteomic shifts align with the observed capacity of VX-445 to mitigate oxidative stress. Specifically, cells upregulate oxidoreductase enzymes in response to TG insult, whereas subsequent treatment with the corrector downregulates their expression. In the list of proteins referred to as the * segment, there are also two hits involved in Ca^2+^ release processes, namely A-kinase anchor protein 5 (Uniprot code P24588) and the ADP-ribosyl cyclase/cyclic ADP-ribose hydrolase 2 (Uniprot code Q10588). Finally, Cytochrome C (Uniprot code P9999) was found to be overexpressed after TG treatment (abundance of 149.5—see Data Availability) and was slightly reduced by Vx-445 (abundance of 87.1—see Data Availability). The cluster and Gene Ontology analyses of the proteins belonging to the ** segment (Figure 5C,D) revealed the presence of many hits belonging to the ribosome machinery (Figure 5C in blue and Figure 5D) and several hits whose molecular function is related to RNA binding (Figure 5C in red and Figure 5D).

These findings align with the established effect of TG injury, which characteristically attenuates or halts de novo protein synthesis to mitigate the accumulation of misfolded polypeptides within the lumen [28]. Crucially, this translational arrest is effectively counteracted by VX-445 administration, demonstrating the capacity of the corrector to rescue cellular biosynthetic machinery under proteotoxic stress conditions.

### 3.5. Identification of Vx-445/Protein Interaction in SH-SY5Y Cell Lysates by DARTS

To identify the direct intracellular protein targets of Vx-445 and investigate its CFTR-independent mechanism of action under ER stress conditions, we performed a Drug Affinity Responsive Target Stability (DARTS) assay. DARTS is a functional proteomics approach aimed at identifying small-molecule binding targets (and off-targets) within complex protein mixtures, such as cell proteomes, without requiring chemical modification of the studied compounds. This quick and straightforward method relies on the increased stability of a target protein against limited proteolysis by a non-specific protease (e.g., subtilisin) upon interaction with small molecules.

**Figure 6:**
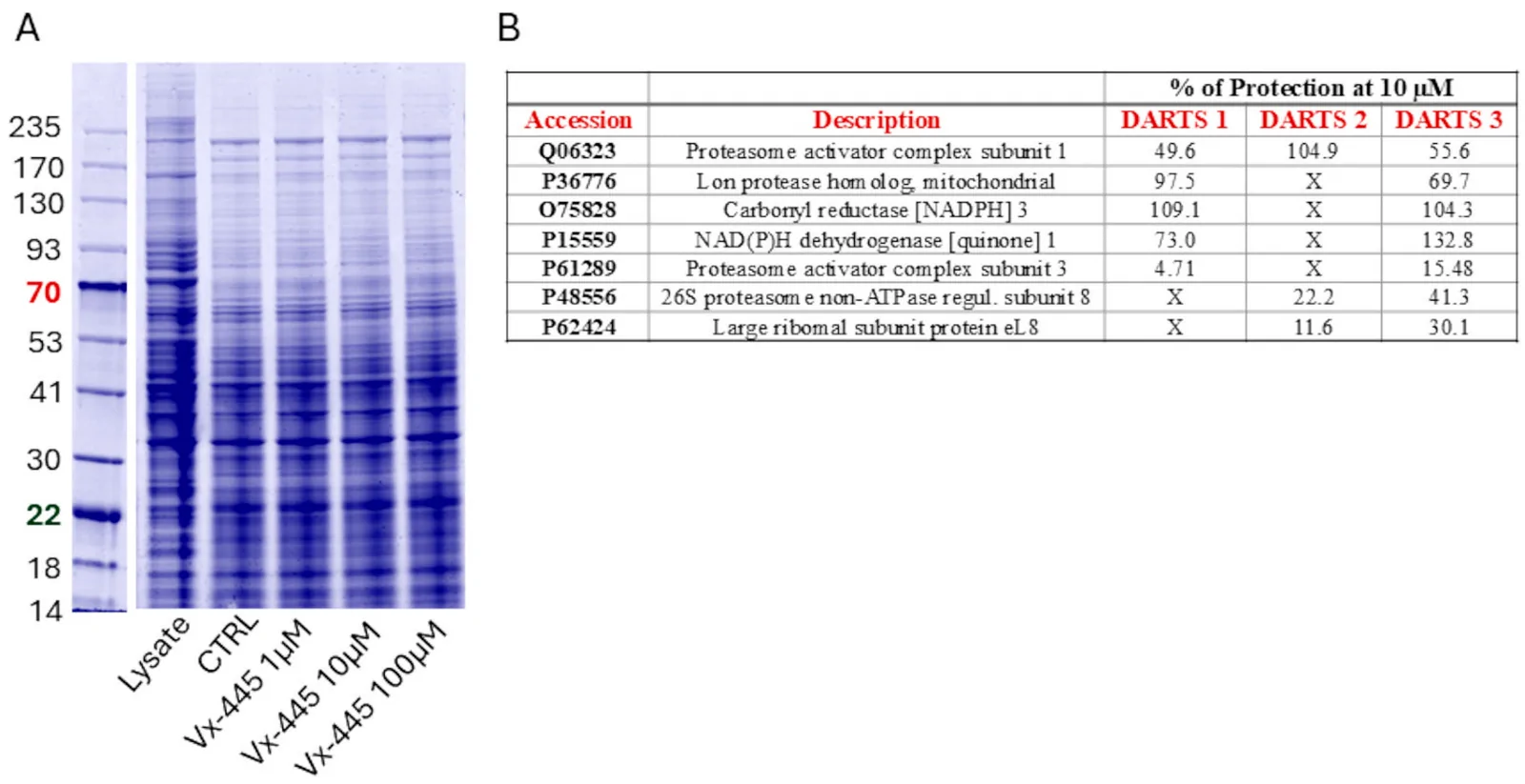
Functional proteomic screening of the VX-445 intracellular interactome via DARTS. (**A**) Representative SDS-PAGE profile stained with Coomassie Brilliant Blue demonstrating dose-dependent target protection against limited subtilisin proteolysis. (**B**) Filtered list of candidates for VX-445 binding targets, indexed by their official UniProt accession number, descriptive protein annotation, and relative magnitude of proteolytic protection expressed as a percentage (%).

In our experimental design, the complete proteome of SH-SY5Y cells was extracted using mild lysis conditions to keep macromolecular complexes intact. Cell lysates were then incubated either with vehicle (DMSO) or titrated concentrations of Vx-445 (1 µM, 10 µM, and 100 µM), followed by controlled digestion using subtilisin; one untreated and undigested sample was kept as a positive control. The proteolytic mixtures were subsequently resolved via SDS-PAGE. Upon Coomassie Brilliant Blue staining, a concentration-dependent protection of specific protein bands against subtilisin cleavage was observed in the Vx-445-treated samples compared to the vehicle control (Figure 6A). This selective protection indicates successful ligand-induced thermodynamic stabilization of target candidates.

Subsequently, the gel lanes were cut into bands and subjected to in situ trypsin digestion to generate peptide mixtures for nano UHPLC-MS/MS analysis. Proteome Discoverer software was used for both protein identification and quantification. To assess the degree of protection against proteolysis afforded by Vx-445, the abundance ratios of proteins between treated and untreated samples were compared. To ensure statistical stringency, only proteins displaying a sample/control ratio ≥ 1.5 in at least two out of three independent biological replicates were considered putative Vx-445 interactors. Applying these strict filtration criteria resulted in a refined final list of approximately 30 candidate hits (Table S1; see Data Availability).

Label-free quantitative analysis revealed a restricted cohort of candidate target proteins that exhibited statistically significant (*p* < 0.05), concentration-dependent protection upon Vx-445 engagement. Notably, among the top-ranking protected hits, we identified key molecular players that directly match the phenotypic and expression proteomics data described above. Specifically, the DARTS approach confirmed the direct binding and stabilization of crucial components of the intracellular ion homeostasis apparatus, strongly aligning with the observed modulation of intracellular Ca^2+^ dynamics and the previous identification of AKAP5 and BST-1/ADP-ribosyl cyclase 2 in the expression proteomics screening. Furthermore, consistent with the pharmacochaperone activity of the molecule, several enzymes residing within the ER network displayed distinct structural protection, thereby corroborating the ability of Vx-445 to physically interact with the proteostasis machinery to reduce misfolding stress.

## 4. Discussion

The ER serves as the primary compartment where secretory and membrane proteins are synthesized and appropriately modified. Proteostasis is a complex and finely regulated mechanism [2]; however, genetic mutations, epigenetic modifications, environmental stress, and aging can disrupt protein processing, leading to ER stress and contributing to the development of many diseases [4]. Cells respond immediately to such stress by activating key UPR mediators to restore homeostasis. Nevertheless, under conditions of chronic or unmitigated ER stress, this adaptive capacity is overwhelmed, and cells undergo apoptosis. Consequently, prolonged ER stress and impaired UPR signaling are increasingly recognized as significant factors across a wide spectrum of human disorders, including PMDs.

Among these is cystic fibrosis (CF), in which mutations in the CFTR gene provoke structural misfolding of the corresponding protein, compromising its function at target sites and thus causing typical CF symptoms. Currently, CF treatment relies on CFTR modulators—small molecules that interact with the mutated protein to improve its processing or function.

Over time, these drugs have demonstrated a broader therapeutic potential beyond their primary clinical indication. Specifically, Pecoraro and colleagues [18,19] showed that the corrector Vx-809 exerts a broader effect by reducing cellular stress in A549 and A375 cell lines. Furthermore, this compound displayed distinct neuroprotective effects evaluated in SH-SY5Y neuroblastoma cells, a well-established in vitro model for neurodegenerative disorders.

Building on this evidence, the aim of the present study was to investigate whether Vx-445, a next-generation corrector currently included as a core component of the triple-combination therapy for CF [22], exhibits similar pleiotropic activity. Specifically, we examined the capacity of Vx-445 to counteract TG-induced ER stress in SH-SY5Y cells. To delineate its molecular mechanisms, we systematically evaluated quantitative changes in the cellular proteome and identified direct intracellular targets of the drug through a DARTS-based functional proteomics approach.

A major finding of this study is the capacity of Vx-445 to modulate ROS levels. Maintaining physiological ROS concentrations, often conceptualized as “beneficial stress”, is critical for cellular homeostasis, as they drive key biochemical processes, including carboxylation, hydroxylation, peroxidation, and the modulation of signaling pathways essential for cell adhesion, differentiation, proliferation, and immune responses. Protein folding and intracellular ROS generation are closely linked; when mitochondrial activity, NADPH oxidase function, or exogenous factors like radiation, drugs, diet, and smoking [29] disrupt the balance between ROS production and clearance, the finely tuned oxidative environment of the ER breaks down. This imbalance results in protein misfolding and accumulation, accelerating ER stress.

In the present study, we demonstrated that Vx-445 markedly reduces ROS production following TG-induced ER stress at multiple time points, with effects detectable by flow cytometry as early as 15 min. This underscores both the dynamic nature of redox balance and the exquisite sensitivity of neuronal cells to oxidative stress. Our proteomics data strongly corroborate this antioxidant capacity: clustering and Gene Ontology analyses revealed that TG upregulates several oxidoreductase enzymes, an effect reversed by Vx-445 treatment. Furthermore, DARTS experiments confirmed a direct interaction between Vx-445 and key redox-related enzymes, including carbonyl reductase [NADPH]_3_, NAD(P)H quinone dehydrogenase 1, and mitochondrial Lon protease. Interruption of this vicious cycle between ER and oxidative stress represents a significant therapeutic advance, as this reciprocal crosstalk constitutes a central driver underpinning PMDs.

Given that intracellular Ca^2+^ stores tightly regulate cellular redox balance by modulating ROS production and clearance, our findings underscore the strict interconnection linking ER stress, oxidative stress, and Ca^2+^ dysregulation.

Across the lifespan of a cell, fluctuations in intracellular Ca^2+^ concentrations mediate essential biological functions, including gene expression, neurotransmitter release, proliferation, and apoptosis. As the principal intracellular Ca^2+^ reservoir, the ER plays a central role while ensuring proper protein folding via Ca^2+^-dependent chaperones and oxidoreductases, such as calreticulin, BiP, calnexin, and members of the thioredoxin family protein disulfide isomerases (PDIs).

Utilizing the fluorescent probe FURA-2AM, we observed that SH-SY5Y cells exposed to TG showed a markedly reduced Δ increase in cytoplasmic Ca^2+^ upon subsequent stimulation, confirming that most luminal Ca^2+^ had already been depleted. In contrast, cells pretreated with Vx-445 for 24 h displayed a significantly higher Δ increase, demonstrating that the drug helps maintain or restore Ca^2+^ homeostasis under stress conditions. Proteomics analyses corroborated these functional biophysical data, revealing significant changes in Ca^2+^ exchange-related proteins. Most notably, we identified significant alterations in A-kinase anchor protein 5 (AKAP5). ER stress triggers Ca^2+^ release through channels such as inositol 1,4,5-trisphosphate receptors (IP_3_Rs) and ryanodine receptors (RyRs). Protein kinase A (PKA) critically regulates IP_3_R1 by sensitizing the channel to IP_3_ through phosphorylation of cytosolic serine residues [30], a process where the AKAP family is essential to target PKA close to its substrates and amplify cAMP-driven signaling [31]. Additionally, proteomic analysis identified cyclic ADP-ribose cyclase/hydrolase 2 as another Ca^2+^-regulating target; its expression was significantly downregulated following TG treatment but was fully restored to baseline levels upon VX-445 administration. Beyond Ca^2+^ handling, this enzyme regulates the second messenger, cyclic ADP-ribose (cADPR), which acts on ryanodine receptors by catalyzing both its synthesis from NAD^+^ and its hydrolysis to ADPR [32], thereby linking ER Ca^2+^ signaling to broader cellular regulatory mechanisms.

The complexity of these interactions underscores how the dysregulation of Ca^2+^ signaling underpins the pathogenesis of multiple human diseases, particularly neurodegenerative disorders. In these contexts, our results demonstrate that the corrector Vx-445 significantly reduces cytoplasmic Ca^2+^ concentrations following TG-induced ER stress, suggesting its potential role in restoring Ca^2+^ homeostasis under conditions of cellular stress.

Using the fluorescent probe FURA-2AM, SH-SY5Y cells exposed to TG showed a markedly reduced Δ increase in cytoplasmic Ca^2+^, indicating that most Ca^2+^ had already been released at the time of measurement. In contrast, cells treated with Vx-445 for 24 h displayed a significantly higher Δ increase, suggesting that the drug helps restore Ca^2+^ homeostasis. Proteomics analyses further support this role, as changes were observed in Ca^2+^ exchange-related proteins, including A-kinase anchor protein 5 (AKAP5). ER stress is known to trigger Ca^2+^ release through channels such as inositol 1,4,5-trisphosphate receptors (IP_3_Rs) and ryanodine receptors (RyRs). The three IP_3_R isoforms exhibit distinct tissue-specific expression patterns, and their activation depends on IP_3_. Among these, protein kinase A (PKA) critically regulates IP_3_R1 by sensitizing its channels to IP_3_ through phosphorylation of cytosolic serine residues [30]. Within this pathway, the AKAP family is essential for targeting PKA to define subcellular sites and for bringing the holoenzyme close to its substrates, thereby amplifying cAMP-driven signaling [31]. Proteomic analysis further identified cyclic ADP-ribose cyclase/hydrolase 2 as another Ca^2+^-regulating protein. Its expression was significantly downregulated following TG treatment but was fully rescued to baseline levels upon Vx-445 administration. Beyond modulating Ca^2+^ homeostasis, this enzyme regulates the second messenger cyclic ADP-ribose (cADPR), which acts through ryanodine receptors. It catalyzes both the synthesis of cADPR from NAD^+^ and its hydrolysis to ADPR [32], linking ER Ca^2+^ signaling to broader cellular regulatory mechanisms.

Beyond Ca^2+^dysregulation, growing evidence indicates that ER stress resulting from aberrant protein accumulation directly impairs mitochondrial function. Since mitochondria are crucial for ROS generation, intracellular signaling, and the regulation of neuronal survival [33], their structural and functional breakdown represents a critical pathogenic event. Under physiological conditions, cytochrome c resides in the mitochondrial intermembrane and intercristae spaces, functioning as an electron carrier in the respiratory chain and binding cardiolipin. Proapoptotic stimuli, including unresolved ER stress, permeabilize the outer mitochondrial membrane and disrupt intercompartmental communication. This structural collapse triggers cytochrome c release into the cytosol, where it activates apoptotic proteases [34]. Given the contribution of apoptosis to neurodegeneration, targeting this pathway represents a promising neuroprotective strategy [35]. In this study, we demonstrated that Vx-445 significantly reduces cytosolic cytochrome c levels following ER stress induction. This finding indicates that the corrector possesses a novel pharmacodynamic capacity to intercept and modulate the mitochondrial apoptotic pathways driven by the accumulation of misfolded proteins. Concurrently, our proteomics data revealed numerous hits associated with ribosomal machinery and RNA molecular functions. This finding aligns with the well-known translational arrest caused by TG: exposure of neuronal cells to TG induces a complete suppression of protein synthesis and the disaggregation of polyribosomes, indicating an inhibition of the translation initiation phase [28]. Here, we demonstrate that the administration of the corrector Vx-445 effectively rescues these alterations, restoring physiological conditions, as illustrated by the heat map in Figure 4.

In conclusion, our findings demonstrate that Vx-445 partially counteracts TG-induced proteomic alterations, highlighting its therapeutic potential to modulate ER stress-related pathways. Taken together, these data indicate that Vx-445 rescues key cellular processes disrupted by ER stress, most notably oxidative stress, Ca^2+^ homeostasis, proteostasis, translational machinery, and mitochondrial function. Although further studies are required to establish its translational clinical relevance in non-CF models, the present work provides a valuable exploratory framework and a solid foundation for future research into the expanded, off-label biological activities of CFTR correctors. Therefore, these results provide a robust mechanistic foundation for the pharmacological repositioning of a clinical compound to treat diverse human PMDs

## Data Availability

DARTS proteomics data are available in the *Mendeley Data* repository at the following DOIs: https://doi.org/10.17632/2wkjsrcjds.1 (DARTS 1), https://doi.org/10.17632/vbvh95ft8t.1 (DARTS 2) and https://doi.org/10.17632/mfpdj9ncf2.1 (DARTS 3). Expression proteomics data are available in the *Mendeley Data* repository at the following DOIs: https://doi.org/10.17632/vt63yhkdj9.1, https://doi.org/10.17632/hnrrpyyrvz.1 and https://doi.org/10.17632/p5wy5bpkr2.1. Furthermore, at the DOI https://doi.org/10.17632/vt63yhkdj9.1, an xls. file is available, showing the * and ** cluster protein abundances in each sample.

## Supplementary Materials

The following supporting information can be downloaded at: https://www.mdpi.com/article/10.3390/cimb48080772/s1.
