# Supplementary material for: Functional Proteomics and Biological Screening of Protein Misfolding Under ER Stress Conditions in a Neuroblastoma Cell Model
Source: Curr Issues Mol Biol. 2026 Jul 29;48(8):772. doi: 10.3390/cimb48080772 (PMC13510333; doi:10.3390/cimb48080772)
Supplement: Supplementary file 1 [file cimb-48-00772-s001.zip › cimb-4453954-supplementary.pdf]

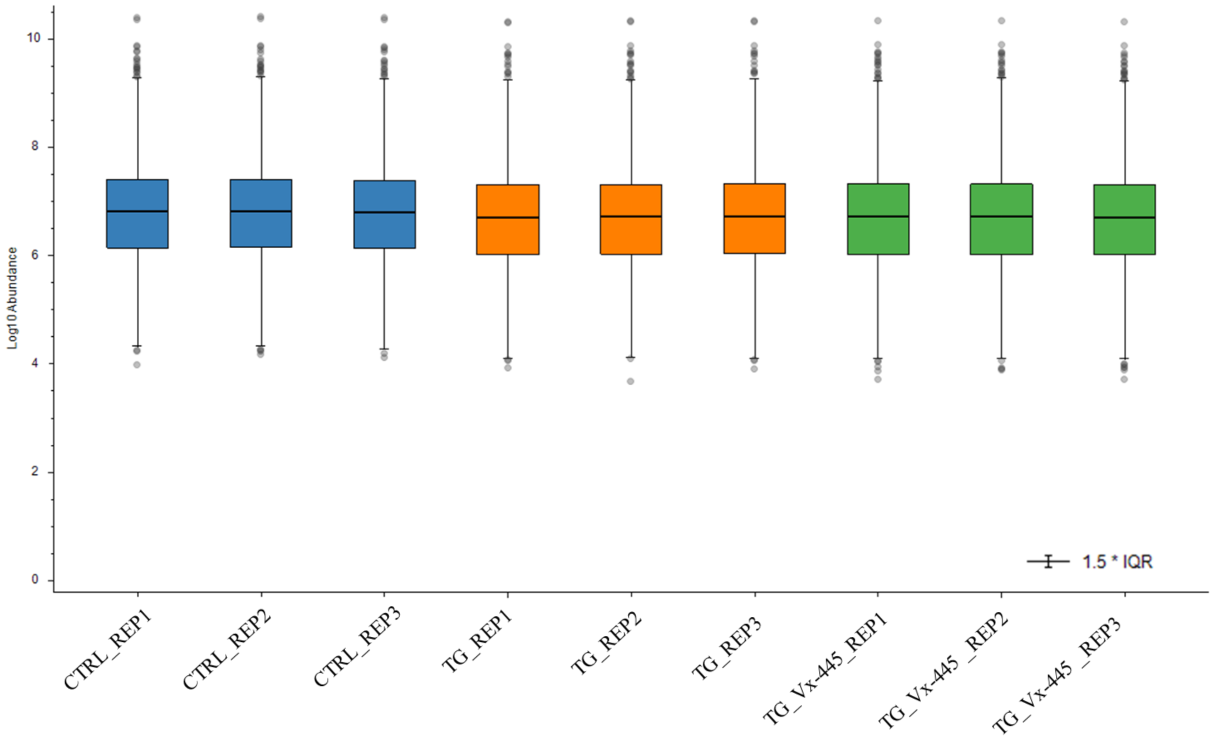

Figure S1: The box-plot reports the proteins abundance of each sample treated with and without TG and Vx-445**.**

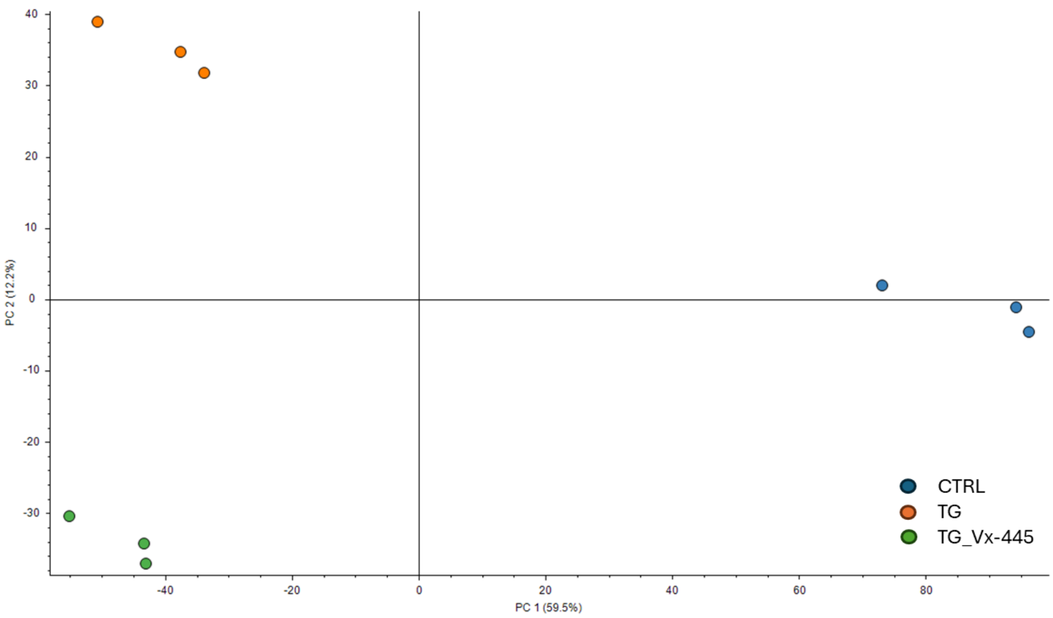

Figure S2: Principal component analysis (PCA) plot is reported using the Scores Plot. Circles represent grouped samples treated with and without TG and Vx-445. Both the x axis and y axis display principal components together with their percentage values.

**Accession**	**Description**	**MW [kDa]**	**DARTS 1**	**DARTS 2**	**DARTS 3**
**Q06323**	Proteasome activator complex subunit 1	28,7	√	√	√
**P36776**	Lon protease homolog, mitochondrial	106,4	√	X	√
**Q9Y6Y8**	SEC23-interacting protein	111	√	X	√
**Q9NX05**	Coactivator of PPAR-γ-like protein 2	120,5	√	X	√
**Q15018**	BRISC complex subunit Abraxas 2	46,9	√	X	√
**Q96FZ2**	Abasic site processing protein HMCES	40,5	√	X	√
**Q9Y295**	Development-regulated GTP binding protein 1	40,5	√	X	√
**Q13011**	Δ(3,5)-Δ(2,4)-dienoyl-CoA isomerase	35,8	√	X	√
**P05141**	ADP/ATP translocase 2	32,8	√	X	√
**P46926**	Glucamine-6-phphate isomerase 1	32,6	√	X	√
**Q96CN7**	Isochorismatase domain-containing protein 1	32,2	√	X	√
**O75828**	Carbonyl reductase [NADPH] 3	30,8	√	X	√
**P15559**	NAD(P)H dehydrogenase [quinone] 1	30,8	√	X	√
**O95865**	DimethylArg dimethylaminohydrolase 2	29,6	√	X	√
**P61289**	Proteasome activator complex subunit 3	29,5	√	X	√
**Q92688**	Acidic leucine-rich nuclear phospho 32B	28,8	√	X	√
**P12004**	Proliferating cell nuclear antigen	28,8	√	X	√
**P39687**	Acidic leucine-rich nuclear phospho 32 A	28,6	√	X	√
**P42345**	Serine/threonine-protein kinase mTOR	288,7	X	√	√
**Q659C4**	La-related protein 1B	105,3	X	√	√
**Q9H7Z7**	Prtaglandin E synthase 2	41,9	X	√	√
**Q8IVD9**	NudC domain-containing protein 3	40,8	X	√	√
**P48556**	26S proteasome non-ATPase regul. subunit 8	39,6	X	√	√
**Q86X76**	Deaminated glutathione amidase	35,9	X	√	√
**P42574**	Caspase-3	31,6	X	√	√
**P62424**	Large ribomal subunit protein eL8	30	X	√	√
**Q01081**	Splicing factor U2AF 35 kDa subunit	27,9	X	√	√

Table S1: Filtered list of Vx-445 expected targets, reported with their accession number, description and molecular weight. √ is reported if the protein has been protected in the relative DARTS experiment.
